# A Comprehensive Analysis of the Role of PAX9 in Head and Neck Squamous Cell Carcinoma

**DOI:** 10.2174/0115665240328109241205084841

**Published:** 2025-01-15

**Authors:** Lang Zeng, Wenjing Yun, Wen-long Luo

**Affiliations:** 1 Department of Otorhinolaryngology, the Second Affiliated Hospital of Chongqing Medical University, Chongqing, China

**Keywords:** PAX9, pan-cancer analysis, HNSCC, apoptosis, tumor suppressor, overall survival (OS)

## Abstract

**Background:**

Paired box 9 (PAX9) has been linked to several human disorders; however, its relevance in Head And Neck Squamous Cell Carcinoma (HNSCC) remains unknown.

**Methods:**

The difference in PAX9 mRNA expression in pan-cancer was analyzed utilizing The Cancer Genome Atlas (TCGA), and the level of PAX9 protein expression across various types of cancer was assessed utilizing the Human Protein Atlas (HPA) and UALCAN databases, as well as the cellular localization of PAX9. UALCAN studied the methylation levels of PAX9 in pan-cancer. The predictive significance of PAX9 in pan-cancer was assessed utilizing the Kaplan-Meier Plotter website. Functional enrichment analysis was carried out with the “cluster Profiler” program. By employing CCK8 and colony formation methods, the influence of PAX9 on the growth of HNSCC cells was evaluated. By conducting a transwell experiment, we assessed the influence of PAX9 on the migration of HNSCC cells. Western blotting was used to determine the levels of Bax and Bcl-2, two proteins involved in the regulation of apoptosis. A nude mouse model was established to study the impact of PAX9 overexpression on the growth of subcutaneous HNSCC tumors.

**Results:**

In HNSCC, the expression of PAX9 was found to be low, while levels of promoter methylation rose considerably. Low PAX9 expression has been linked to a decrease in overall survival (OS) rates among individuals with HNSCC. Furthermore, overexpressing the PAX9 gene decreased HNSCC cell proliferation, migration, and invasion while boosting apoptosis rates.

**Conclusion:**

The abnormal expression of PAX9 is linked to various cancers. In HNSCC, PAX9 is a potential tumor suppressor, inhibiting tumor invasion and migration. The results reveal a potentially significant new therapeutic target for HNSCC.

## 1 INTRODUCTION

HNSCC encompasses squamous cell carcinomas affecting the hypopharynx, larynx, nasopharynx, oro-pharynx, and oral cavity [1], which is the most common malignant tumor in the head and neck region, ranking seventh in terms of frequency [2]. Common risk factors associated with HNSCC include tobacco smoking, heavy alcohol consumption, betel nut chewing, and infection with human papillomavirus (HPV) [3-5]. The biological behavior, clinical symptoms, therapeutic response, and prognosis of HNSCC demonstrate great variety [6, 7], whereas the highly invasive nature of HNSCC impedes any improvement in overall survival rates for patients [8-10]. As a result, learning more about the etiology and pathogenesis of HNSCC, as well as discovering enhanced biomarkers and molecular targets for therapeutic interventions in the diagnosis and prognostic evaluation, will have a major clinical impact on improving the prognosis.

PAX9 encodes a family of transcription factors [11, 12], which are essential for embryonic development and organogenesis [13, 14], specifically tooth initiation and differentiation [15, 16]. Importantly, studies have shown a strong link between abnormal PAX9 expression and proliferation, apoptosis, invasion, and metastasis of lung cancer [17], esophageal cancer [18], ovarian cancer [19], and cervical cancer [20]. However, the particular carcinogenic mechanism of PAX9 in malignant tumors remains unknown, and there is a scarcity of research on PAX9's function in the onset and development of HNSCC.

The current study aims to enhance the investigation of expression levels and their prognostic significance of PAX9 in pan-cancer by utilizing public databases, followed by an analysis of its methylation level, immu-noregulation, and tumorigenic signaling pathway, spe-cifically in HNSCC. Furthermore, focusing on HNSCC as the research subject, we conducted preliminary investigations into the impact of the overexpression of PAX9 on tumor formation, proliferation, migration and invasion ability. This study establishes a theoretical foundation for further exploration of PAX9 in HNSCC.

## 2 MATERIALS AND METHODS

### 2.1 Data Collection

Clinical data and mRNA expression profiles from TCGA and the University of California Santa Cruz (UCSC) Xena were acquired to assess PAX9 expression in pan-cancer. The original data was standardized using the transcripts per million (TPM) method. Subsequently, a log2(TPM+1) transformation was applied for more in-depth analysis [21].

### 2.2 Expression Analysis of PAX9 and Survival Analysis

The expression levels of PAX9 mRNA in 33 tumors and their corresponding normal tissues were compared and analyzed using mRNA expression profiling. The PAX9 protein expression level in tumors was determined by analyzing the HPA database (http://www.proteinatlas.org) [22] and the UALCAN database (https://ualcan.path.uab.edu/index.html) [23]. Additionally, the subcellular localization of the PAX9 protein and the immunohistochemical level of HNSCC were obtained from HPA [22].

The prognostic value of PAX9 expression on different types of cancer was evaluated utilizing the Mantel-Cox test in GEPIA2.0 [24]. Additionally, the Kaplan-Meier plotter (http://kmplot.com/analysis) was utilized to investigate the interaction between tumor survival and PAX9 expression using log-rank testing [25].

### 2.3 Promoter Methylation of PAX9 in HNSCC

In this study, we used UALCAN, which provides the TCGA RNA sequencing database of 31 different types of cancer and clinical data, to examine the methylation levels of PAX9 in HNSCC [23].

### 2.4 Immune Infiltration and Functional Enrichment Analysis

The patients were allocated into two different groups based on a 50% cut-off value in the study. The ssGSEA algorithm [12], available in the R package GSVA [1.46.0], was utilized for the calculation of immune cell infiltration using 24 markers provided by Bindea [13].

We employed the cluster Profiler package for enrichment analysis [14]. The z-score value corresponding to each enrichment entry was calculated using the provided molecule values in the GO plot package [15]. Ultimately, the ggplot2 package was employed to represent the data visually.

### 2.5 Cell Culture and Materials

HNSCC cell line (SNU1076) was obtained from Zhejiang Meisen Cell Technology Co., LTD, and cultured in RPMI-1640 media (G4535, Servicebio, China) with 10% fetal bovine serum (C04001, Viva Cell, Israel) and 1% of 100U/mL penicillin-streptomycin solution (C0222, Beyotime, China) at 37°C with 5% CO_2_.

### 2.6 Establishment of a Cell Line Overexpressing PAX9

SNU1076 was separated into two groups: vector (transfected with a control lentiviral vector from Fenghui Biotechnology in Hunan Province, China) and PAX9 overexpression. In 6-well plates, SNU1076 cells in optimum growth conditions were planted 7.5×10^4^ cells/well. While the cell grew to 30% of the dish, the lentivirus transfected HNSCC cells at a MOI of 50. To improve transfection efficiency, polybrene (C0351, Beyotime, China) (5ug/ml) was added. After 24h of lentivirus transfection, the petri dishes were replaced with fresh media and replenished daily thereafter. Puromycin Dihydrochloride (ST551, Beyotime, China) (5ug/ml) was added after 72h of transfection, resulting in about 70% transfection efficiency under fluorescence microscopy. After two rounds of screening, the puromycin concentration was reduced by half, and selection was maintained to acquire stable cell lines for future research.

### 2.7 Western Blotting

Proteins were obtained from SNU1076 cells. Then, we measured the protein concentration with a BCA protein concentration determination kit (P0010, Beyotime, China). The electrophoresis technique was used to separate proteins (30 μg) and subsequently, the protein was transferred onto a 0.22 μm PVDF membrane (88518, Thermo Fisher Scientific, China). The membrane was then sealed in a rapid sealing solution (P0252, Beyotime, China) for 15 min at ambient temperature. The cells were incubated overnight at 4°C with anti-PAX9 antibody (1:500; #AG2804, Beyotime), anti-Bax antibody (1:500; #WL01637, Wan Lei Biotech), anti-Bcl-2 antibody (1:500; #WL01556, Wan Lei Biotech), and anti-β-actin antibody (1:2000; #R23613 Zenbio). Then, at ambient temperature, the membrane was incubated with the secondary antibody (#A0208, Beyotime) for 2h. Finally, the protein was developed using enhanced chemiluminescence (ECL; P0018AS; Beyotime). The relative protein expression level was standardized to that of β-actin and quantified utilizing the ImageJ software.

### 2.8 Colony Formation Assay and Cell Counting Kit-8 Assay

The overexpression group and the vector group were separated into 5 experimental groups based on different lengths of time in culture, specifically the 24-h group, 48-h group, 72-h group, 96-h group, and 120-h group. A 96-well plate was used to seed cells (8×10^3^ cells/well) for incubation. Then, at each respective time point of 24 h, 48 h, 72 h, 96 h, and 120 h, CCK-8 (C0041; Beyotime; China) was included in the corresponding groups with a volume of 10 μl per well. After a four-hour incubation period at an incubator set to a temperature of 37 °C, the absorbance value at 450 nm was detected by Spectra Max iD5 (Molecular Devices).

### 2.9 Invasion and Migration Assay

The HNSCC cells’ migratory ability was evaluated through wound-healing experiments. Initially, a 6-well plate was used to seed the cells (1×10^6^ cells/well). After 24h, the scratch area was observed and compared by culturing the cells in a serum-free medium for another 24h. Transwell analysis was utilized to determine vertical migration and invasion capacity. For the migratory experiment, we seeded 2×10^5^ cells into the upper chamber with an 8um pore membrane using a volume of 200ul, while the lower chamber was filled with 600ul complete medium with 20% fetal bovine serum. After incubation for 24h, the migrated cells were fixed, stained, photographed and quantified. To evaluate the invasion capability, a gel matrix (C0372; Beyotime; China) was applied onto the transwell membrane prior to conducting procedures similar to those used in the migration assay.

### 2.10 Construction of Xenograft Models on Nude Mice

Ten 4-week-old BALB/c-NU mice, sourced from Changzhou Cavens Laboratory Animal Center Co., Ltd), were allocated into two groups at random and raised in a controlled environment free from pathogens. The experimental group (SNU1076 cells transfected with a lentiviral vector overexpressing PAX9) and the control group (SNU1076 cells transfected with a control vector) were inoculated subcutaneously into nude mice at a concentration of 1×10^7^ cells per mouse (150μl cell suspension) to establish a xenograft tumor model. Tumor size was measured weekly after subcutaneous tumor formation, and tumor volume was calculated to generate a tumor growth curve. Immunohistochemical analysis was performed to assess the expression of Ki-67, Bax, and Bcl-2 in collected tumor tissues after four weeks.

### 2.11 Statistical Analysis

The data were analyzed using the SPSS software (SPSS, Inc., Chicago, IL, USA). The data were statistically evaluated using a t-test and were reported as the mean ± standard deviation (SD). The statistical significance of the observed disparity is indicated by a p-value lower than 0.05, suggesting a meaningful and impactful difference within the data [26-28].

## 3 RESULTS

### 3.1 Pan-cancer PAX9 Expression Analysis

**Fig. (1):**
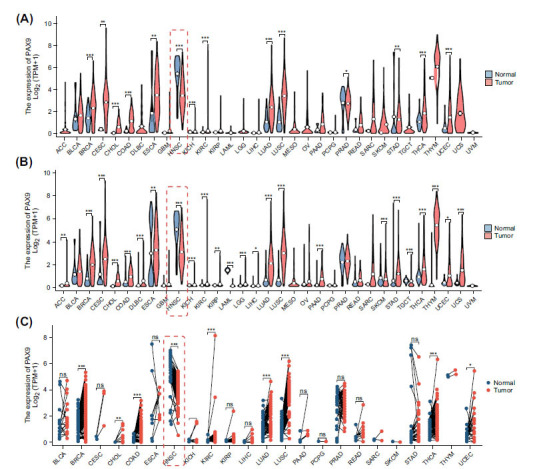
Pan-cancer PAX9 expression analysis. (**A**) Comparative analysis of PAX9 differential expression in pan-cancer (TCGA dataset). (**B**) Comprehensive analysis of PAX9 differential expression in pan-cancer (TCGA+GTEx datasets). (**C**) Paired sample analysis revealing the differential expression of PAX9 in pan-cancer.

PAX9 mRNA expression levels were significantly different among a wide variety of tumors in the TCGA database (Fig. **1**). Thereinto, PAX9 levels were upregulated in breast invasive carcinoma (BRCA), cervical squamous cell carcinoma and endocervical adenocarcinoma (CESC), cholangiocarcinoma (CHOL), colon adenocarcinoma (COAD), esophageal carcinoma (ESCA), lung adenocarcinoma (LUAD), lung squamous cell carcinoma (LUSC), thyroid carcinoma (THCA), thymoma (THYM) and, uterine corpus endometrial carcinoma (UCEC), while HNSCC、prostate adeno-carcinoma (PRAD) and stomach adenocarcinoma (STAD) exhibited significantly low expression levels. (Fig. **1A**) Furthermore, when comparing the corresponding normal tissues in the UCSC database with tumor samples, similar results were observed for the PAX9 mRNA expression levels (Fig. **1B**). Notably, significant differences were also observed in paired tumor samples, and HNSCC showed significantly lower expression (Fig. **1C**). The findings suggested that PAX9 plays a significant part in various tumors. However, further investigation is necessary to elucidate its specific functions.

### 3.2 The Association Between PAX9 Expression and Prognosis

**Fig. (2):**
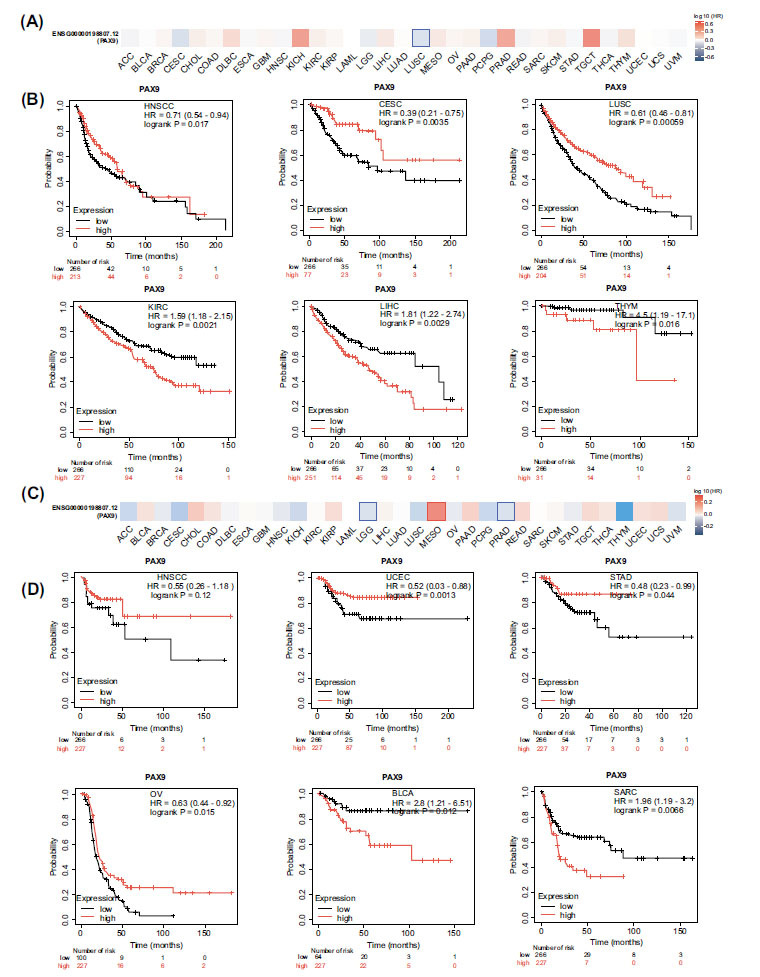
The association between PAX9 expression and cancer patient prognosis. (**A**) The PAX9 OS Analysis in pan-cancer (TCGA). (**B**) The impact of PAX9 on the prognostic OS of various types of tumors. (**C**) The role of PAX9 in RFS Analysis (TCGA) across various types of cancer. (**D**) The influence of PAX9 on the prognosis of recurrence-free survival in various types of tumors.

The influence of the expression of PAX9 on the prognosis remains unclear. The findings from this study demonstrate that PAX9 exhibits distinct prognostic significance across different tumor types (Fig. **2**). Specifically, PAX9 expression is inversely linked to overall survival (OS) prognosis in HNSCC, whereas it is not correlated with OS prognosis in HNSCC (Figs. **2A** and **2B**). Furthermore, we analyzed the influence of PAX9 expression on recurrence-free survival (RFS) among tumor patients, revealing a positive correlation between PAX9 expression and RFS prognosis in UCEC, STAD, and ovarian carcinoma (OV). Conversely, a negative association was observed between PAX9 expression and RFS prognosis in Bladder Urothelial Carcinoma (BLCA), and Sarcoma (SARC) (Figs. **2C** and **2D**). However, further research is imperative to enhance our comprehension of the molecular mechanisms by which PAX9 operates in tumors.

### 3.3 The Expression and Functional Analysis of PAX9 in HNSCC

**Fig. (3):**
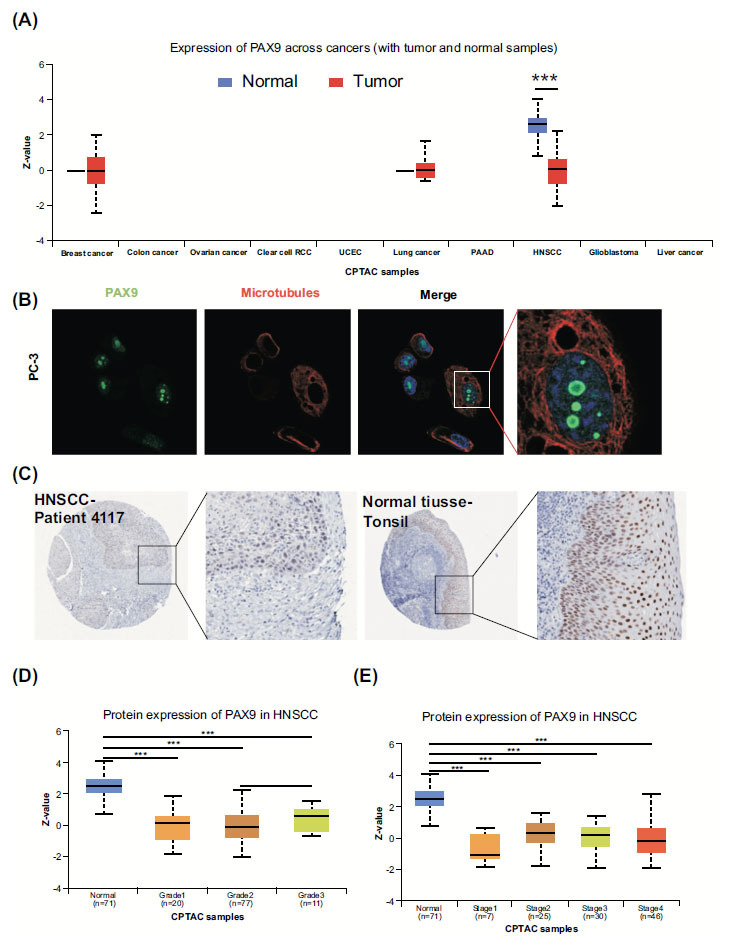
Analysis of PAX9 protein expression in HNSCC. (**A**) Distinctive protein expression of PAX9 was observed in CPTAC samples. (**B**) Immunocytochemistry for detecting the subcellular location of PAX9 in PC-3 cell line. PAX9 localized to the nucleoli (green). Microtubules were stained in red. The antibody of PAX9 was HPA001572. (**C**) Immunohistochemical analysis of PAX9 expression in HNSCC tumor tissues and normal tonsil tissues. (**D**) Expression levels of PAX9 in various grades of HNSCC. (**E**). Expression levels of PAX9 across various stages of HNSCC.

Upon further analysis, the expression of PAX9 protein was decreased in HNSCC tissues, while no substantial differences were observed in other tumor types or between normal tissues. Furthermore, the PAX9 protein was predominantly localized within the nucleus of squamous epithelial cells (Fig. **3B**). Consistent with the mRNA expression patterns, low or negligible levels of PAX9 protein were detected in HNSCC, whereas significantly higher expression was observed in squamous epithelial cells of normal tonsil tissues (Fig. **3C**). Regrettably, there were no significant variances in PAX9 expression observed among patients with varying grades and stages of HNSCC, according to the 8th Edition of the AJCC Cancer Staging Manual [29-31] (Figs. **3D** and **3E**). Therefore, the results concluded that PAX9 plays a crucial part in the initiation and advancement of HNSCC.

**Fig. (4):**
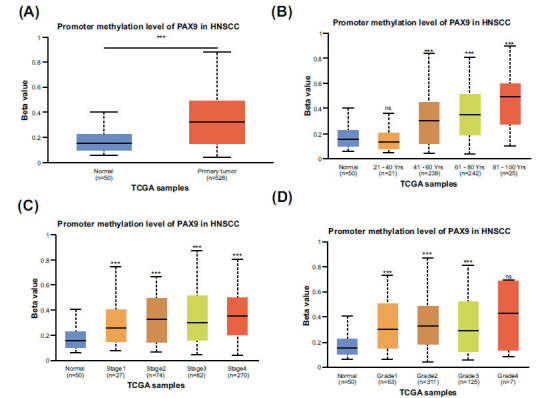
Methylation levels of PAX9 in HNSCC. (**A**) The methylation level of PAX9 in HNSCC (TCGA). (**B**) Methylation levels of PAX9 across different age cohorts in HNSCC. (**C**) The methylation level of PAX9 in various stages of HNSCC. (**D**) PAX9 methylation levels in various grades of HNSCC.

Simultaneously, the analysis results indicated a significantly higher methylation level of the PAX9 promoter in HNSCC compared to the normal group (Fig. **4A**), concurrently exhibiting a clear age-related increasing trend (Fig. **4B**). Although no statistically significant variances were detected in the methylation levels of PAX9 among various stages and grades, it was notably elevated in patients with grade 4 HNSCC (Figs. **4C** and **4D**). These findings further support the notion that DNA methylation acts a crucial part in the progression of HNSCC.

**Fig. (5):**
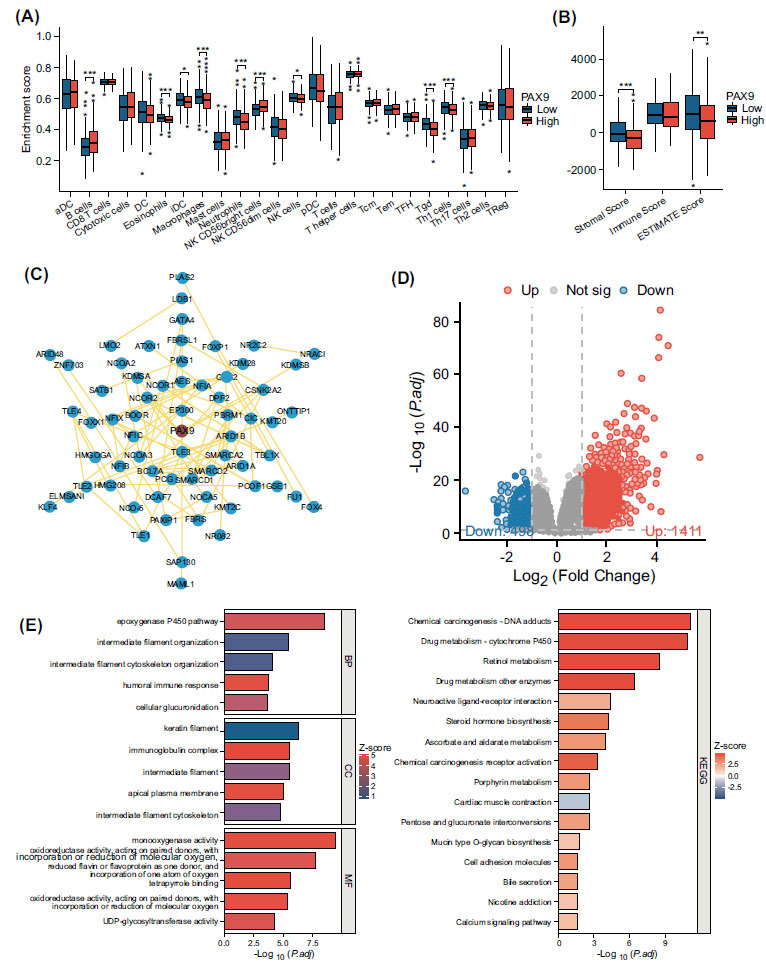
Function analysis of PAX9 in HNSCC. (**A**) Difference analysis of various immune cell infiltrations in HNSCC. (**B**) Difference analysis of immune scores in HNSCC. (**C**) Genes associated with PAX9 (BIOGRID, minimum evidence=4). (**D**) Volcano map E of genes in the high-low PAX9 expression group in HNSCC. (**E**) GO-KEGG analysis of differential genes.

Although preliminary research has indicated the significance of PAX9 in tumors, its specific role in HNSCC remains unclear. Given the remarkable advan-cements in cancer treatment through immunotherapy and the influence of the immune microenvironment on tumor progression, this study investigated the Infiltration of immune cells between the high and low-expression groups of PAX9. The results revealed a notable decrease in immune cell infiltration in the high expression group, primarily involving immature dendritic cells (iDCs), macrophages, neutrophils, natural killer cells (NK cells), gamma delta T cells (T gd cells), and helper T1 cells (Th1 cells) (Fig. **5A**). In addition, higher Stromal Score and ESTIMATE Score values were found in the low expression group of PAX9; however, no statistical difference was found for Immune Score (Fig. **5B**). These findings indicate that PAX9 plays a critical part in regulating the tumor microenvironment of HNSCC.

The protein network associated with PAX9 is revealed in Fig. **5C**. A volcano plot displaying differentially expressed genes is shown in Fig. **5D**. Further screening for genes exhibiting |LogFC| > 1 and p < 0.05 revealed enrichment of differential gene ontology (GO) terms primarily involved in the epoxygenase P450 pathway, intermediate filament organization, immunoglobulin complex, monooxy-genase activity, etc. Additionally, the KEGG analysis demonstrated the enrichment of pathways like chemical carcinogenesis of DNA adducts and drug metabolism-cytochrome P450 (Fig. **5E**). These findings suggest that PAX9 potentially regulates various signaling pathways in HNSCC and provides potential targets for therapeutic intervention.

### 3.4 Effect of PAX9 Overexpression on HNSCC Cells

**Fig. (6):**
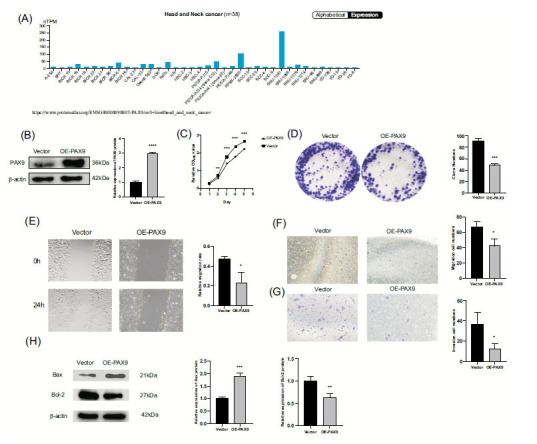
PAX9 expression level of HNSCC cell line and the effect of PAX9 on SNU1076 cells. (**A**) PAX9 expression level of HNSCC cell line. (**B**) The verification outcome following the overexpression of PAX9 in SNU1076. (**C**) Effect of overexpression of PAX9 on proliferation of SNU1076 cells. (**D**) Effect of overexpression of PAX9 on cloning of SNU1076 cells. (**E**) Effect of PAX9 overexpression on migration ability of SNU1076 cells for 24h. (**F**) Effect of overexpression of pax9 on migration ability of SNU1076 cells. (**G**) Effect of overexpression of PAX9 on invasion ability of SNU1076 cells. (**H**) Expression levels of Bax and Bcl-2 proteins in SNU1076 cells after overexpression of PAX9.

To further investigate the specific influence of PAX9 in HNSCC, we selected the SNU1076 cell line based on its relatively low expression of PAX9 compared to other HNSCC cell lines (Fig. **6A**) [22]. First, we successfully established a stable overexpression of PAX9 in SNU1076 cells, which was verified through Western blot analysis (Fig. **6B**). The proliferative ability of cells in the PAX9 overexpression group was significantly attenuated (Fig. **6C**), and their clonogenic formation capacity was markedly reduced (Fig. **6D**). Additionally, both migration ability (Figs. **6E** and **6F**) and invasion capability (Fig. **6G**) were significantly decreased in cells overexpressing PAX9. These findings suggest that PAX9 may function as a gene with suppressive effects in HNSCC and participate in the process of tumor proliferation, migration and invasion. Western blot analysis indicated an increase in the protein Bax and a decrease in the protein Bcl-2 in the SNU1076 overexpression group. (Fig. **6H**), indicating that PAX9 may promote apoptosis in SNU1076 cells. The findings of this study illustrate that PAX9 exerts inhibitory effects on the proliferation, migration, and invasion of SNU1076 cells while also simultaneously promoting apoptosis in these cells. However, it is necessary to research the concrete mechanisms involved further.

### 3.5 Animal Experiment

**Fig. (7):**
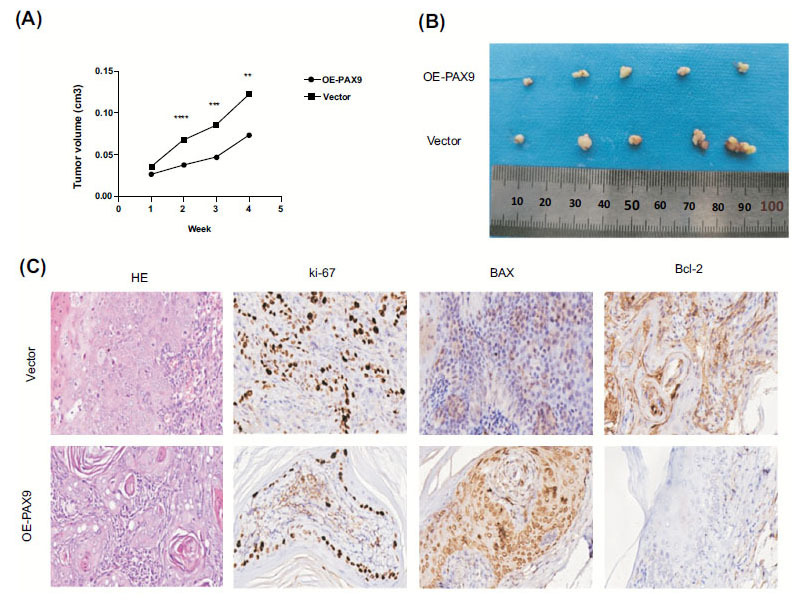
The impact of PAX9 on tumor formation in nude mice. (**A**) The influence of PAX9 overexpression on the growth of subcutaneously transplanted tumors in nude mice. (**B**) Representative graph depicting tumor size (taken 4 weeks post-tumor formation). (**C**) Representative graph illustrating HE, Ki67, Bax, and Bcl-2 staining patterns in tumor tissue (40×).

A subcutaneous tumor transplantation model was established in nude mice to observe further the impact of PAX9 on tumor formation *in vivo*. The findings demonstrated a significant deceleration in tumor growth in the PAX9 overexpression group, with statistically significant differences (Figs. **7A** and **7B**). Moreover, the ki-67 expression level was decreased in tumor tissues from the overexpression group (Fig. **7C**), which aligns with the results obtained from *in vitro* experiments. Moreover, Bax expression increased, and Bcl-2 expression decreased in tumor tissues from the overexpression group (Fig. **7C**). *In vivo* experiments also demonstrated that PAX9 exerted inhibitory effects on tumor cell proliferation and facilitated the induction of tumor cell apoptosis.

## 4 DISCUSSION

In recent years, treatment modalities have been remarkably advanced for HNSCC, including surgical interventions, cytotoxic chemotherapy, radiation therapy, targeted molecular therapy, and innovative immunotherapy techniques [29-36]. These advancements are due to the impressive progress in medical technology. However, because HNSCC is highly heterogeneous and invasive, the majority of patients receive a diagnosis at an advanced stage and require a combination of surgical intervention and chemoradiotherapy [37, 38]. Regrettably, patients in the advanced stages of the disease have a bleak outlook, underscoring the necessity of investigating precise treatment methods such as molecular targeted therapy and immunotherapy instead of relying solely on palliative care [39]. Consequently, there is an immediate requirement to pinpoint sensitive biomarkers and specific biological markers for early detection, treatment assessment, and clinical prognosis evaluation of HNSCC. PAX9 encodes a transcription factor, which plays a pivotal part in diverse biological processes, encompassing regulating gene expression, precise cell migration and differentiation, as well as controlling cell proliferation and programmed cell death. [40-43]. Furthermore, there have been observations of PAX9 dysregulation in various cancerous tumors. For example, research has shown that the down-regulation of PAX9 expression is linked to human oral-esophageal squamous cell carcinoma (OESCC), and this decrease is connected to alcohol consumption and promoter hypermethylation [44]. Exposure to ethanol inhibits the NOTCH signaling pathway, resulting in a reduction of PAX9 expression in esophageal squamous epithelial cells [18]. The expression of PAX9 is diminished within cervical cancer tissues, resulting in the attenuation of cervical cancer cell proliferation and the facilitation of cellular apoptosis. Additionally, it is associated with clinical pathological features and prognosis [20]. These findings indicate that PAX9 could play a potential part in cervical cancer. However, the specific functional mechanisms of its role are still not fully clear. As a result, this research utilized an extensive bioinformatic analysis of PAX9 across various public databases.

Initially, the study observed notable differences in the expression of PAX9 mRNA levels in paired samples of pan-cancer and notably reduced expression in HNSCC within the TCGA database. Furthermore, our findings indicated a negative correlation between PAX9 and overall survival rates across multiple tumor types. Identifying aberrant expression of a gene across various malignant tumors may present it as a potential novel biomarker for early cancer screening, diagnosis, treatment, and prognosis [45].

Additionally, we explored the potential mechanisms by which PAX9 may impact the development and advancement of HNSCC. There are evidence indicating that the tumor microenvironment (TME) exerts a pivotal influence on the biological mechanisms driving tumorigenesis. As a result, there has been an increasing focus on researching the TME when studying malignant tumors [46-49]. The current research found a significant link between PAX9 expression and Stromal Score and ESTIMATE Score in HNSCC. Following this, an analysis was carried out to understand the important immune cell components in HNSCC. The findings revealed a significant contrary relevance in the infiltration of immature dendritic cells (iDC), macrophages, neutrophils, natural killer (NK) cells, T gd cells, and type 1 helper T cells (Th1) [50, 51]. For example, dendritic cells (DCs) have the ability to promote immune tolerance, and iDCs have stronger tolerogenic properties. As DCs mature, their immune tolerance changes into immunogenicity. Therefore, it is crucial to maintain an immature state or inhibit DC maturation in order to induce immune tolerance [52-54]. The inverse relationship between PAX9 and iDC in the tumor microenvironment implies that PAX9 could have a substantial impact on the advancement and outlook of HNSCC by regulating the tumor microenvironment.

To further elucidate the underlying mechanisms of HNSCC, our study conducted pathway enrichment analysis to go deep into the oncogenic function of PAX9. The differentially expressed genes exhibited prominent enrichment in pathways related to the epoxygenase P450 pathway, intermediate filament organization, immunoglobulin complex, and monooxygenase activity, according to GO analysis. An analysis using the KEGG database revealed a notable enrichment in pathways associated with chemical carcinogenesis, DNA adducts, and drug metabolism involving cytochrome P450. Importantly, the epoxygenase P450 pathway is a biological process responsible for oxidizing arachidonic acid to produce biologically active leukotrienes that act as crucial regulatory parts in various biological processes, such as promoting neovascularization and regulating tumor cell proliferation and migration [55, 56]. Consequently, the analysis of enrichment indicates that PAX9 may play a pivotal part in HNSCC by modulating the epoxygenase P450 signaling pathway. The findings from KEGG enrichment indicate that PAX9 could have significant biological functions through the pathway of DNA adducts in chemical carcinogenesis. Chemical carcinogens can cause damage to DNA, and this damaged DNA can undergo genetic mutations during replication, which occur at the earliest stages of cancer initiation. As such, the outcomes of the pathway enrichment analysis imply that PAX9 may play a pivotal part in various signaling pathways associated with the biological processes of HNSCC [57-59], subsequently leading to alterations in protein expression encoded by genes, resulting in changes in biological processes. A gene mutation can lead to the emergence of clonal cell populations that exhibit clear proliferative advantages, thereby instigating the development of human cancer [60-62]. The findings from the pathway enrichment analysis indicate that PAX9 could be involved in various signaling pathways related to the biological functions of HNSCC.

Moreover, we conducted some experiments on cellular models to detect the effect of PAX9 on the SNU1076 cell line, which exhibited diminished levels of PAX9. The results found that PAX9 upregulated the expression of Bax in HNSCC cellular models [63-65]. However, Bcl-2 did the opposite. Furthermore, results from immunohistochemistry tests on subcutaneous tumor xenografts in nude mice revealed similar results, indicating that PAX9 can promote the apoptosis of tumor cells. Invasion and spread are essential biological traits of cancerous tumors that contribute to the poor prognosis seen in HNSCC [3]. Metastasis is a common occurrence in the initial phases of cancer development, but the symptoms of metastasis usually become apparent after a number of years [66-69]. Consequently, the results from both the wound healing and transwell assays indicated that the heightened expression of PAX9 substantially hindered the migratory and invasive capabilities of SNU1076 cells. This suggests that the overexpression of PAX9 suppresses the invasion and migration abilities of SNU1076 cells. The cellular experiments mentioned above provide evidence supporting a tumor-suppressing role for PAX9, which aligns with our bioinformatics analysis showing decreased levels of PAX9 in HNSCC and a negative association between PAX9 expression and prognosis in HNSCC. Therefore, these findings indicate that PAX9 may effectively inhibit tumor growth by significantly reducing the proliferation, migration, and invasion of HNSCC cells while promoting their apoptosis. This potential mechanism appears to be closely associated with the abnormal methylation of the PAX9 promoter [70-72], which can only be further explored in the follow-up research due to the limited time and funds.

## CONCLUSION

In brief, this study employed bioinformatics analysis methods to examine the expression profiles and clinical prognostic significance of PAX9 in pan-cancer. Additionally, we examined the methylation levels, immune regulation, and tumorigenic signaling pathways of PAX9 in HNSCC. Finally, we undertook an initial inquiry into the impact of PAX9 on the biological functionality of HNSCC cell lines. The discoveries indicate that PAX9 may serve as a tumor suppressor gene in HNSCC and contribute to tumor initiation and advancement. This research lays a theoretical foundation for potential treatments for HNSCC.

## Data Availability

The data and supportive information are available within the article.

## LIST OF ABBREVIATIONS

HNSCC: Head and neck squamous cell carcinoma
PAX9: Paired box 9
TCGA: The Cancer Genome Atlas
OS: Overall survival
RFS: Recurrence-free survival

## References

[1] Johnson D.E., Burtness B., Leemans C.R., Lui V.W.Y., Bauman J.E., Grandis J.R. (2020). Head and neck squamous cell carcinoma.. Nat. Rev. Dis. Primers.

[2] Jayawickrama S.M., Ranaweera P.M., Pradeep R. (2024). Developments and future prospects of personalized medicine in head and neck squamous cell carcinoma diagnoses and treatments.. Cancer Reports.

[3] Saada-Bouzid E., Peyrade F., Guigay J. (2019). Molecular genetics of head and neck squamous cell carcinoma.. Curr. Opin. Oncol..

[4] Solomon B., Young R.J., Rischin D. (2018). Head and neck squamous cell carcinoma: Genomics and emerging biomarkers for immunomodulatory cancer treatments.. Semin. Cancer Biol..

[5] Morse D.E., Psoter W.J., Cleveland D. (2007). Smoking and drinking in relation to oral cancer and oral epithelial dysplasia.. Cancer Causes Control.

[6] Tanadini-Lang S., Balermpas P., Guckenberger M. (2020). Radiomic biomarkers for head and neck squamous cell carcinoma.. Strahlenther. Onkol..

[7] Leemans C.R., Snijders P.J.F., Brakenhoff R.H. (2018). The molecular landscape of head and neck cancer.. Nat. Rev. Cancer.

[8] Liang F., Wang R., Du Q., Zhu S. (2021). An epithelial–mesenchymal transition hallmark gene-based risk score system in head and neck squamous-cell carcinoma.. Int. J. Gen. Med..

[9] Affolter A., Lammert A., Kern J., Scherl C., Rotter N. (2021). Precision medicine gains momentum: Novel 3D models and stem cell-based approaches in head and neck cancer.. Front. Cell Dev. Biol..

[10] Gong S.Q., Xu M., Xiang M.L., Shan Y.M., Zhang H. (2019). The expression and effection of MicroRNA-499a in high-tobacco exposed head and neck squamous cell carcinoma: A bioinformatic analysis.. Front. Oncol..

[11] Chen X., Li Y., Paiboonrungruang C. (2022). PAX9 in cancer development.. Int. J. Mol. Sci..

[12] Liu J., Yang M., Su M. (2022). FOXG1 sequentially orchestrates subtype specification of postmitotic cortical projection neurons.. Sci. Adv..

[13] Li R., Chen Z., Yu Q., Weng M., Chen Z. (2019). The function and regulatory network of Pax9 gene in palate development.. J. Dent. Res..

[14] Bhol C.S., Patil S., Sahu B.B., Patra S.K., Bhutia S.K. (2021). The clinical significance and correlative signaling pathways of paired box gene 9 in development and carcinogenesis.. Biochim. Biophys. Acta Rev. Cancer.

[15] Bonczek O., Balcar V.J., Šerý O. (2017). >PAX9 gene mutations and tooth agenesis: A review.. Clin. Genet..

[16] Šerý O., Bonczek O., Hloušková A. (2015). A screen of a large Czech cohort of oligodontia patients implicates a novel mutation in the PAX9 gene.. Eur. J. Oral Sci..

[17] Zhao Z., Szczepanski A.P., Tsuboyama N. (2021). PAX9 determines epigenetic state transition and cell fate in cancer.. Cancer Res..

[18] Shi M., Ren S., Chen H. (2021). Alcohol drinking inhibits NOTCH – PAX9 signaling in esophageal squamous epithelial cells.. J. Pathol..

[19] Soto J.A., Rodríguez-Antolín C., Vera O. (2021). Transcriptional epigenetic regulation of Fkbp1/Pax9 genes is associated with impaired sensitivity to platinum treatment in ovarian cancer.. Clin. Epigenetics.

[20] Liu J., Wang Y.Q., Niu H.B., Zhang C.X. (2022). PAX9 functions as a tumor suppressor gene for cervical cancer via modulating cell proliferation and apoptosis.. Kaohsiung J. Med. Sci..

[21] Tan L., Li W., Su Q. (2023). The comprehensive analysis of the prognostic and functional role of N-terminal methyltransferases 1 in pan-cancer.. PeerJ.

[22] Colwill K., Gräslund S., Graslund S. (2011). A roadmap to generate renewable protein binders to the human proteome.. Nat. Methods.

[23] Chandrashekar D.S., Karthikeyan S.K., Korla P.K. (2022). UALCAN: An update to the integrated cancer data analysis platform.. Neoplasia.

[24] Tang Z., Kang B., Li C., Chen T., Zhang Z. (2019). GEPIA2: an enhanced web server for large-scale expression profiling and interactive analysis.. Nucleic Acids Res..

[25] Nagy Á., Munkácsy G., Győrffy B. (2021). Pancancer survival analysis of cancer hallmark genes.. Sci. Rep..

[26] Razavi Vakhshourpour S., Nateghpour M., Shahrokhi N., Motevalli Haghi A., Mohebali M., Hanifian H. (2022). Potential of RH5 antisense on Plasmodium falciparum proliferation abatement.. Iran. J. Parasitol..

[27] Badri M., Ghaffarifar F., Hassan Z.M., Dalimi A., Cortes H. (2020). Immunoregulatory effects of somatic extract of Toxocara canis on airway inflammations in murine model.. Iran. J. Parasitol..

[28] Cui Z., Guo Y., Zhou Y. (2020). Transcriptomic analysis of the developmental similarities and differences between the native retina and retinal organoids.. Invest. Ophthalmol. Vis. Sci..

[29] Doescher J., Veit J.A., Hoffmann T.K. The 8th edition of the AJCC Cancer Staging Manual : Updates in otorhinolaryngology, head and neck surgery. HNO 2017.

[30] Lydiatt W.M., Patel S.G., O’Sullivan B. (2017). Head and Neck cancers-major changes in the American Joint Committee on cancer eighth edition cancer staging manual. CA Cancer J Clin.

[31] Glastonbury C.M. (2020). Critical changes in the staging of head and neck cancer.. Radiol. Imaging Cancer.

[32] Burtness B., Harrington K.J., Greil R. (2019). Pembrolizumab alone or with chemotherapy versus cetuximab with chemotherapy for recurrent or metastatic squamous cell carcinoma of the head and neck (KEYNOTE-048): a randomised, open-label, phase 3 study.. Lancet.

[33] Seiwert T.Y., Burtness B., Mehra R. (2016). Safety and clinical activity of pembrolizumab for treatment of recurrent or metastatic squamous cell carcinoma of the head and neck (KEYNOTE-012): an open-label, multicentre, phase 1b trial.. Lancet Oncol..

[34] Ferris R.L., Blumenschein G., Fayette J. (2016). Nivolumab for recurrent squamous-cell carcinoma of the head and neck.. N. Engl. J. Med..

[35] Mehanna H., Robinson M., Hartley A. (2019). Radiotherapy plus cisplatin or cetuximab in low-risk human papillomavirus-positive oropharyngeal cancer (De-ESCALaTE HPV): an open-label randomised controlled phase 3 trial.. Lancet.

[36] Gillison M.L., Trotti A.M., Harris J. (2019). Radiotherapy plus cetuximab or cisplatin in human papillomavirus-positive oropharyngeal cancer (NRG Oncology RTOG 1016): a randomised, multicentre, non-inferiority trial.. Lancet.

[37] Wang C.W., Biswas P.K., Islam A., Chen M.K., Chueh P.J. (2024). The use of immune regulation in treating Head and Neck Squamous Cell Carcinoma (HNSCC).. Cells.

[38] Hashim D., Genden E., Posner M., Hashibe M., Boffetta P. (2019). Head and neck cancer prevention: from primary prevention to impact of clinicians on reducing burden.. Ann. Oncol..

[39] Newman J.G., Hall M.A., Kurley S.J. (2021). Adjuvant therapy for high-risk cutaneous squamous cell carcinoma: 10-year review.. Head Neck.

[40] Chi N., Epstein J.A. (2002). Getting your Pax straight: Pax proteins in development and disease.. Trends Genet..

[41] Kendall J., Liu Q., Bakleh A. (2007). Oncogenic cooperation and coamplification of developmental transcription factor genes in lung cancer.. Proc. Natl. Acad. Sci. USA.

[42] Sánchez R.S., Sánchez S.S. (2013). Characterization of pax1, pax9, and uncx sclerotomal genes during Xenopus laevis embryogenesis.. Dev. Dyn..

[43] Short S., Holland L.Z. (2008). The evolution of alternative splicing in the Pax family: the view from the Basal chordate amphioxus.. J. Mol. Evol..

[44] Xiong Z., Ren S., Chen H. (2018). PAX9 regulates squamous cell differentiation and carcinogenesis in the oro‐oesophageal epithelium.. J. Pathol..

[45] Manier S., Liu C.J., Avet-Loiseau H. (2017). Prognostic role of circulating exosomal miRNAs in multiple myeloma.. Blood.

[46] Jin M.Z., Jin W.L. (2020). The updated landscape of tumor microenvironment and drug repurposing.. Signal Transduct. Target. Ther..

[47] Zafari N., Khosravi F., Rezaee Z. (2022). The role of the tumor microenvironment in colorectal cancer and the potential therapeutic approaches.. J. Clin. Lab. Anal..

[48] Zhong Z., Vong C.T., Chen F. (2022). Immunomodulatory potential of natural products from herbal medicines as immune checkpoints inhibitors: Helping to fight against cancer via multiple targets.. Med. Res. Rev..

[49] Ferrer G., Álvarez-Errico D., Esteller M. (2022). Biological and molecular factors predicting response to adoptive cell therapies in cancer.. J. Natl. Cancer Inst..

[50] Bruno A., Ferlazzo G., Albini A., Noonan D.M. (2014). A think tank of TINK/TANKs: tumor-infiltrating/tumor-associated natural killer cells in tumor progression and angiogenesis.. J. Natl. Cancer Inst..

[51] O’Melia M.J., Rohner N.A., Manspeaker M.P., Francis D.M., Kissick H.T., Thomas S.N. (2020). Quality of CD8 + T cell immunity evoked in lymph nodes is compartmentalized by route of antigen transport and functional in tumor context.. Sci. Adv..

[52] Sato K., Yamashita N., Yamashita N., Baba M., Matsuyama T. (2003). Regulatory dendritic cells protect mice from murine acute graft-versus-host disease and leukemia relapse.. Immunity.

[53] Song J.K., Yin S.Y., Li W. (2021). An update on the role of long non-coding RNAs in psoriasis.. Chin. Med. J. (Engl.).

[54] Neufeld M.J., Lutzke A., Pratx G., Sun C. (2021). High- Z metal-organic frameworks for x-ray radiation-based cancer theranostics.. Chemistry.

[55] Carpenter R., Brady M.F., Gene B.A.X. (2023). BAX Gene.. In: StatPearls..

[56] Nigam S.K., Bush K.T., Bhatnagar V., Poloyac S.M., Momper J.D. (2020). The systems biology of drug metabolizing enzymes and transporters: Relevance to quantitative systems pharmacology.. Clin. Pharmacol. Ther..

[57] Colin Garner R. (1998). The role of DNA adducts in chemical carcinogenesis.. Mutat. Res..

[58] Allmann S., Mayer L., Olma J. (2020). Benzo[a]pyrene represses DNA repair through altered E2F1/E2F4 function marking an early event in DNA damage-induced cellular senescence.. Nucleic Acids Res..

[59] Luo J., Chen J.W., Zhou J. (2022). TBX20 inhibits colorectal cancer tumorigenesis by impairing NHEJ-mediated DNA repair.. Cancer Sci..

[60] Basu A., Damage D.N.A. (2018). Mutagenesis and Cancer.. Int. J. Mol. Sci..

[61] Khodadadian A., Darzi S., Haghi-Daredeh S. (2020). Genomics and transcriptomics: The powerful technologies in precision medicine.. Int. J. Gen. Med..

[62] Al-Gabri N.A., Saghir S.A.M., Al-Hashedi S.A. (2021). Therapeutic potential of thymoquinone and its nanoformulations in pulmonary injury: A comprehensive review.. Int. J. Nanomedicine.

[63] Liu X., Chu W., Shang S. (2020). Preliminary study on the anti-apoptotic mechanism of Astragaloside IV on radiation-induced brain cells.. Int. J. Immunopathol. Pharmacol..

[64] Dogan S., Mason M.C., Govindaraju A. (2013). Interrelationships between apoptosis and fertility in bull sperm.. J. Reprod. Dev..

[65] Tang D., Zhang S., Shi X. (2019). Combination of astragali polysaccharide and curcumin improves the morphological structure of tumor vessels and induces tumor vascular normalization to inhibit the growth of hepatocellular carcinoma.. Integr. Cancer Ther..

[66] Klein C.A. (2020). Cancer progression and the invisible phase of metastatic colonization.. Nat. Rev. Cancer.

[67] Zhang T., Prasad P., Cai P. (2017). Dual-targeted hybrid nanoparticles of synergistic drugs for treating lung metastases of triple negative breast cancer in mice.. Acta Pharmacol. Sin..

[68] van Attekum M.H.A., Eldering E., Kater A.P. (2017). Chronic lymphocytic leukemia cells are active participants in microenvironmental cross-talk.. Haematologica.

[69] Das S., Sarrou E., Podgrabinska S. (2013). Tumor cell entry into the lymph node is controlled by CCL1 chemokine expressed by lymph node lymphatic sinuses.. J. Exp. Med..

[70] Bai G., Song J., Yuan Y. (2019). Systematic analysis of differentially methylated expressed genes and site-specific methylation as potential prognostic markers in head and neck cancer.. J. Cell. Physiol..

[71] Smetannikova NA, Evdokimov AA, Netesova NA (2019). Application of GLAD-PCR assay for study on DNA methylation in regulatory regions of some tumor-suppressor genes in lung cancer.. Zhongguo Fei Ai Za Zhi.

[72] Rani L., Mathur N., Gupta R. (2017). Genome-wide DNA methylation profiling integrated with gene expression profiling identifies PAX9 as a novel prognostic marker in chronic lymphocytic leukemia.. Clin. Epigenetics.

